# Robust and Hydrophobic Silica/Polyimide Aerogel with Pomegranate-like Structure for Thermal Insulation and Flame Retardancy up to 1300 °C

**DOI:** 10.3390/molecules30081709

**Published:** 2025-04-11

**Authors:** Junyong Chen, Defang Pan

**Affiliations:** School of Chemistry and Chemical Engineering, Qilu Normal University, Jinan 250200, China; pdf5207@126.com

**Keywords:** silica/polyimide, aerogel, pomegranate-like structure, fire-retardant, thermal insulation

## Abstract

The inherent brittleness of silica aerogels has hindered their application in thermal protection systems. To overcome this limitation, we developed a silica/polyimide composite aerogel with a bio-inspired “pomegranate-like” structure through in situ gelation. The strategic integration of polyimide nanofibers into the silica matrix created an interlocking network that immobilized silica particles, effectively resolving the mechanical fragility. By modulating the polyimide precursor (polyamic acid) concentration to 0.08 g/cm^3^ through polyimide nanofiber reinforcement, the compressive strength reached 2.86 MPa—12 times greater than that of unmodified silica aerogel. The material demonstrated multifunctional performance: exceptional flame resistance (withstanding 1300 °C flame for 20 min with self-extinguishing behavior), high hydrophobicity (123° water contact angle), and ultralow thermal conductivity (0.035 W/(m·K)). This synergistic combination of tunable mechanics, thermal stability, and insulation properties positions the composite as an advanced solution for next-generation thermal protection materials.

## 1. Introduction

Silica aerogel, a nanostructured porous material composed of a three-dimensional network of interconnected Si-O-Si bonds, exhibits exceptional porosity (80–99.8%) and ultralow density (0.003–0.5 g/cm^3^). These unique structural characteristics endow the material with unparalleled thermal insulation properties, making it a prime candidate for thermal protection systems in aerospace and extreme environments [1,2,3]. Nevertheless, the intrinsic brittleness of silica aerogels, as documented in recent studies [4,5,6,7], remains a critical bottleneck for practical implementation. This mechanical fragility originates from two fundamental aspects of its architecture: (i) Structurally, the sol–gel-derived network contains weak interfacial interactions (primarily van der Waals forces) between silica nanoparticles, resulting in inadequate load transfer efficiency under stress; (ii) Chemically, the predominantly covalent siloxane (Si-O-Si) bonds lack energy dissipation pathways, rendering the material susceptible to catastrophic crack propagation. These limitations manifest as powderization under compressive loads and delamination in multilayer systems, ultimately restricting its deployment in load-bearing thermal protection scenarios [8,9].

To improve silica aerogels’ mechanical robustness, chemical and physical modification strategies have been developed, including silicon source optimization [10,11,12], organic cross-linking [13,14,15], and fiber reinforcement [16,17]. Organosilane precursors prove particularly effective, where organic-functionalized siloxane structures enhance framework flexibility through covalent integration. The incorporated organic components simultaneously boost hydrophobicity for moisture resistance. Li et al. [18] synthesized crack-free polymethylsilsesquioxane (PMSQ) aerogels via a methyltriethoxysilane (MTES) precursor, using cetyltrimethylammonium bromide (CTAB) to control the MTES phase separation. The resultant material retained hydrophobicity after 400 °C air exposure for an hour. He et al. [19] developed a silica sol-enhanced hybrid system with vinyltrimethoxysilane (VTMS)/vinylmethyldimethoxysilane (VMDMS), where hydroxyl groups from silica sol enabled copolymerized networks through dehydration–condensation. This architecture achieved 80% strain tolerance over 10 compression cycles via ambient drying.

Despite the enhanced flexibility imparted by organosilicon modification, the elevated organic content (typically 25–40 vol%) in silica aerogels markedly degrades their thermal stability (>400 °C) and flame retardancy (LOI < 22%), critically limiting their deployment in thermal protection systems [20]. This inherent limitation underscores the pressing need for methodologies that concurrently improve mechanical resilience and thermal stability. Inorganic dopants, notably graphene [21], carbon nanotubes (CNTs) [22], and ceramic fibers [23], have demonstrated flame-retardant efficacy through radical scavenging and char layer formation mechanisms. Kong et al. [24] pioneered a fiber-reinforced architecture employing methyltriethoxysilane (MTES)-derived matrices infiltrated into ceramic fiber networks via sol–gel impregnation, achieving exceptional thermal stability (900 °C, 1 h) with <3% linear shrinkage. However, interfacial incompatibility between inorganic reinforcements and siloxane matrices often induces particulate detachment during thermal cycling (ΔT > 500 °C), as evidenced by >15% mass loss in combustion tests. The persistent challenge lies in fabricating nanocomposites with covalently interlocked Si-O-C/Si-O-M (M = metal) 3D networks through scalable processing routes while maintaining ultralow density (<0.1 g/cm^3^).

**Figure 1:**
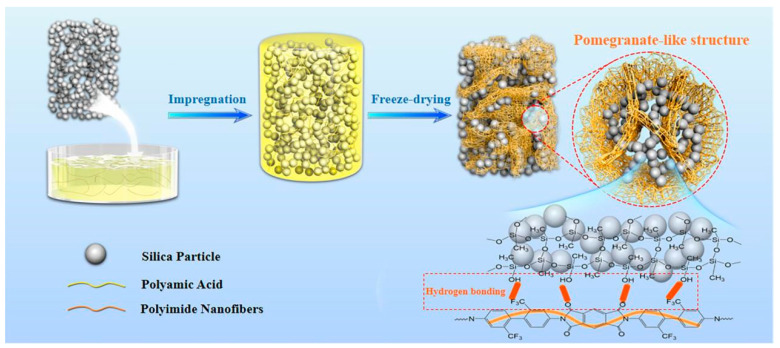
Schematic diagram of the synthesis process for Si/PI aerogel with a “pomegranate-like” structure.

Building upon the fundamental characteristics of polyimide (PI)—a high-performance polymer featuring imide rings in its backbone with exceptional mechanical robustness, thermal stability (>500 °C decomposition temperature), and intrinsic flame retardancy [25,26,27,28]—an innovative silica/PI composite aerogel system was developed through in situ gelation engineering. This strategy successfully constructs a hierarchical “pomegranate-like” architecture where PI nanofibers encapsulate silica particles, as illustrated in Figure 1. The polyimide-formed nanofibers establish a robust anchoring system for silica microspheres through a pomegranate-like structural configuration. This architecture is further reinforced by extensive hydrogen bonding networks at the phase interfaces, which significantly enhance both chemical compatibility and interfacial interactions between components. These synergistic effects collectively resolve the intrinsic brittleness of silica while simultaneously endowing the composite material with exceptional mechanical performance. Crucially, the aerogel maintains outstanding thermal insulation capabilities coupled with remarkable flame retardancy. The material’s superior hydrophobicity ensures reliable performance in high humidity environments. These multifunctional characteristics position this composite aerogel as a promising thermal insulation candidate for aerospace applications, building construction, and industrial thermal management systems.

## 2. Results and Discussion

### 2.1. Microstructure and Composition Analysis

**Figure 2:**
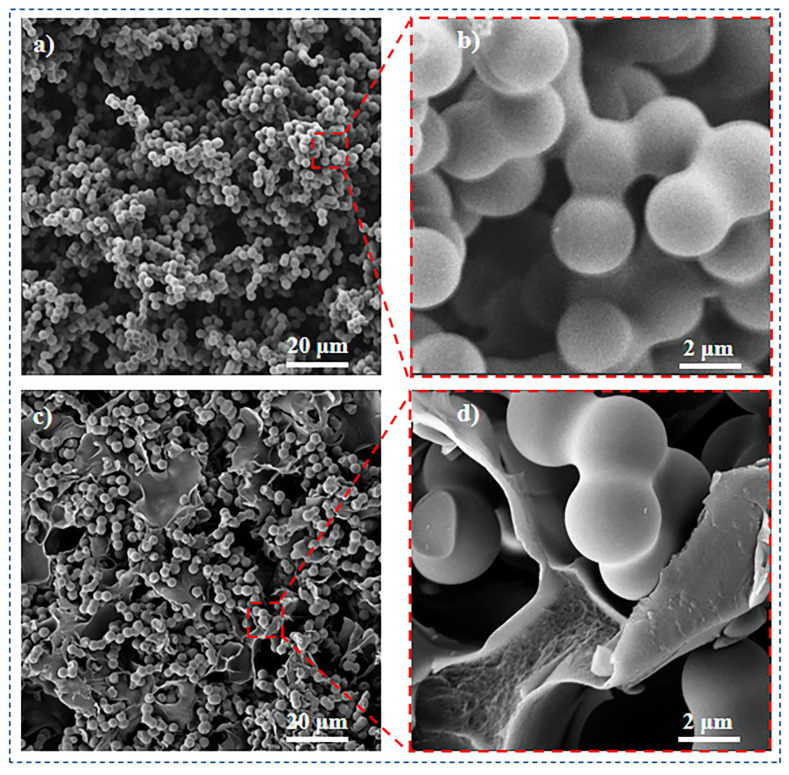
SEM images of samples with different magnifications. (**a**) Si/PI-0 in the scale of 20 μm, (**b**) Si/PI-0 in the scale of 2 μm, (**c**) Si/PI-6 in the scale of 20 μm, and (**d**) Si/PI-6 in the scale of 2 μm.

Firstly, pure silica aerogel (named Si/PI-0) with high porosity was prepared using the sol–gel method (Figure 2a,b). Subsequently, a solution of polyimide precursor (polyamic acid) at a specific concentration was impregnated into the silica pores. Within these pores, the polyamic acid underwent in situ polymerization to form a PI nanofiber gel, ultimately resulting in the composite aerogel Si/PI-6, as depicted in Figure 2c,d. As observed in Figure 2, due to the addition of PI, partial disintegration of silica spheres does occur. This indicates that pure silica aerogel is susceptible to external factors, leading to disintegration, which indirectly highlights its poor mechanical performance and explains its brittleness. The disintegrated silica particles resemble scattered “pomegranate seeds”, while the introduced PI nanofiber layers acts as a robust “pomegranate rind”, firmly encapsulating the silica. Together, they form a “pomegranate-like” encapsulated structure. This “pomegranate-like” structure offers unique advantages: (i) The polyimide nanofibers filling the spaces between the silica particles adhere firmly to the surface of the silica particles, tightly linking the two phases and providing additional skeletal support for the material (Figure S2) [29]; (ii) The PI nanofibers serve as “buffering zones”, facilitating stress release between the silica particles and playing a crucial role in buffering local stress/strain; (iii) At the molecular level, a significant number of hydrogen bonds exist between the polyimide chains and the hydroxyl groups of the silica, further enhancing the interfacial interaction between the two phases and thereby imparting good mechanical integrity to the aerogel [30]. Additionally, by adjusting the concentration of the polyamic acid (PAA) solution, samples with varying PI contents were obtained and labeled as Si/PI-0, Si/PI-2, Si/PI-4, Si/PI-6, and Si/PI-8. As the PAA concentration increases, it is evident that the PI content ultimately retained in the composite aerogel material also increases (Figure S3).

**Figure 3:**
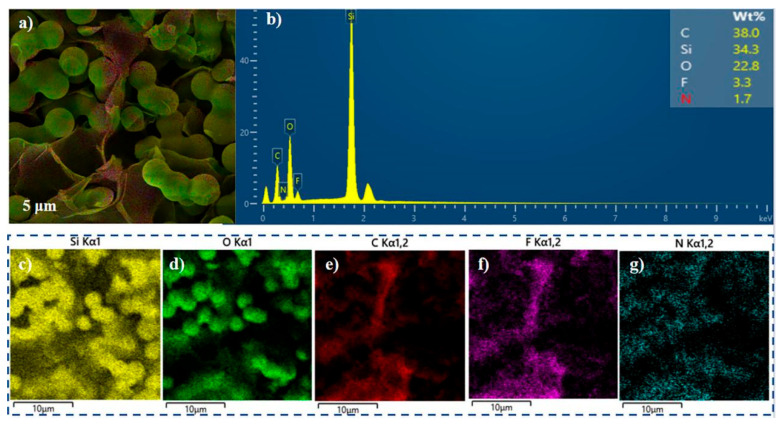
(**a**) SEM image of the Si/PI-6 aerogel. (**b**) Elemental (Si, O, C, F, N) content of the Si/PI-6 aerogel. (**c**–**g**) EDS spectrum of the Si/PI-6 aerogel: (**c**) Si, (**d**) O, (**e**) C, (**f**) F, and (**g**) N.

Energy Dispersive X-ray Spectroscopy (EDS) analysis revealed the elemental distribution on the surface of the composite material. Figure 3a,b shows that sample Si/PI-6 has silicon (Si), oxygen (O), carbon (C), fluorine (F), and nitrogen (N) contents of 33.8%, 30.3%, 38.0%, 3.3%, and 1.7%, respectively, on its surface. Figure 3c,d demonstrates the uniform distribution of Si and O within the silica particles, while carbon (C), fluorine (F), and nitrogen (N) are more concentrated in the PI nanofiber layers, as illustrated in Figure 3e–g. Figure 3 indicates that the spherical silica particles and the laminar polyimide nanofibers have very clear phase interfaces, yet they interpenetrate and are tightly connected, forming a complementary advantage. The surface chemical properties of the composite material were determined by Fourier Transform Infrared (FTIR) spectroscopy. Figure S4 displays the Fourier Transform Infrared Spectra (FTIR) of the silica aerogel and its composites, further confirming the successful introduction of PI nanofibers. The broad bands observed at 1000–1200 cm^−1^ in the spectra of all aerogel composites are attributed to the asymmetric stretching vibration of Si-O-Si [31]. The sharp peak at 765 cm^−1^ corresponds to the asymmetric stretching vibration of Si-C and the wagging vibration of -CH_3_ in the Si-CH_3_ group, while the stretching vibration of C-H in the Si-CH_3_ group is evident in the peak at 1270 cm^−1^ [32]. In the spectra of the composites, the peaks at 1781 cm^−1^ and 1719 cm^−1^ correspond to the symmetric and unsymmetric stretching of the amide group, respectively, indicating the completion of imidization of polyamic acid within the aerogel system. Additionally, the absorption peak of the benzene ring at 1490 cm^−1^ and the stretching vibration absorption peak of the C-N-C bond at 1365 cm^−1^ also confirm the successful introduction of PI [33]. Furthermore, as the amount of PI introduced increases, the absorption peak of the Si-C bending vibration at 850 cm^−1^ gradually weakens until it is completely obscured [34]. In addition, XRD measurements were performed on both the Si/PI-0 sample and the polyimide-doped Si/PI-6 composite, with the results clearly presented in Figure S5. The diffraction patterns for both samples exhibit prominent diffuse scattering peaks within the 5–30° range, which is characteristic of amorphous silica materials [35]. The observation results confirm that the amorphous characteristics of silica remain unaffected by the addition of polyimide. These results clearly demonstrate the successful introduction of PI and its significant increase with the increase in PI content, which is also consistent with the results obtained from SEM characterization.

The brittle silica particle networks are uniformly encapsulated within a polyimide fiber layer through an in situ gelation strategy, resulting in a unique “pomegranate-like” structure. The two-phase system is uniformly composited and tightly interconnected, offering additional skeletal support to the material and significantly aiding in stress release among the silica particles. Furthermore, hydrogen bonding between the silica and PI nanofibers allows for molecular-level interaction between the two phases, achieving a more stable bonding. Consequently, this composite material exhibits superior mechanical strength, flame retardancy, and moisture resistance compared to pure silica aerogels.

### 2.2. Mechanical Properties

Mechanical properties serve as a crucial indicator for evaluating materials and play a vital role in broadening their applications. Due to the brittleness and fragility of silica aerogels, polyimide is incorporated into the silica aerogel via a straightforward impregnation method. When combined, they create a distinctive pomegranate-like structure, which notably improves the mechanical properties of the silica. In this study, the mechanical properties of the samples are characterized using a universal mechanical testing machine.

**Figure 4:**
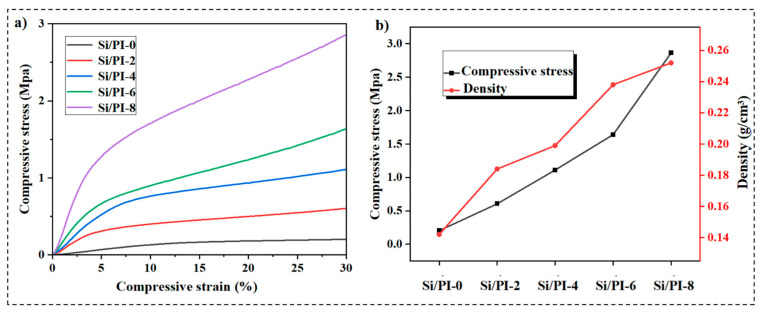
(**a**) Stress–strain (σ–ε) curves; (**b**) density and strength at 60% compression of the samples.

Within the loading range of the samples, two distinctly different regions are observed: one is the linear elastic region, where the strain ε remains below approximately 8%, and the other is the densification region, where the stress increases sharply thereafter. To elucidate the variations scope in the linear elastic regime, a linear fitting analysis was applied to the elastic deformation regions of the stress–strain (σ–ε) curves for all samples, as illustrated in Figure S6. The analysis results reveal that the introduction of polyimide significantly enhances the compressive strength of the material but simultaneously narrows the stress–strain (σ–ε) linearity range from 8% (for Si/PI-0) to 3–4% (from Si/PI-2 to Si/PI-8). In addition, by analyzing the compressive stress–strain (σ–ε) curves of all samples, we determined their yield strengths by the 0.2% offset method, as shown in Figure S7. The yield strength (irreversible point) of Si/PI-0 (without polyimide) occurred at 9.1% strain with a magnitude of 0.125 MPa. In contrast, for all polyimide-modified samples, the yield strength (irreversible point) appeared at <5% strain, with a significant increase in yield strength from 0.27 MPa to 1.06 MPa. This trade-off is attributed to the following mechanisms: The polyimide network reinforces structural integrity, enabling higher stress tolerance but restricting elastic deformation due to the addition of a densified PI nanofiber network. The initial linear elastic region reflects the elastic deformation of the skeleton within the composite aerogel, whereas the subsequent densification region is associated with the elimination of pores and the mutual collision between stacked layers during further loading [36]. The results indicate that the mechanical properties of the composite aerogel were evaluated, revealing that the composite aerogel can be compressed up to 30% without catastrophic collapse. As the PI content increases, the strength of the composite aerogel also increases (Figure 4a). At a strain of 30%, the compressive strength of pure silica is 0.23 MPa, whereas the compressive strength of the composite Si/PI-8 reaches 2.86 MPa, which is more than 12 times higher than that of pure silica aerogel, demonstrating exceptional mechanical properties. As shown in Figure 4b, as the density of the composite increases from 142 kg/m^3^ to 252 kg/m^3^, the compressive strength increases by more than 12 times, illustrating that the introduction of polyimide significantly enhances the mechanical properties of silica. The key factor contributing to the high mechanical properties of the composite aerogel is that the polyimide nanofibers filled between the silica particles provide additional skeletal support and act as a “buffer zone” to dissipate external stress and accommodate large deformations, thereby better transferring forces and facilitating stress release among the silica particles. Additionally, at the molecular scale, a large number of hydrogen bonds exist between the polyimide chains and the hydroxyl groups of silica, further enhancing the interfacial interaction between the two phases and significantly improving the mechanical properties of the material. The exceptional mechanical properties of the composite are crucial for ensuring its comprehensive utilization.

### 2.3. Thermal Insulation Performance

**Figure 5:**
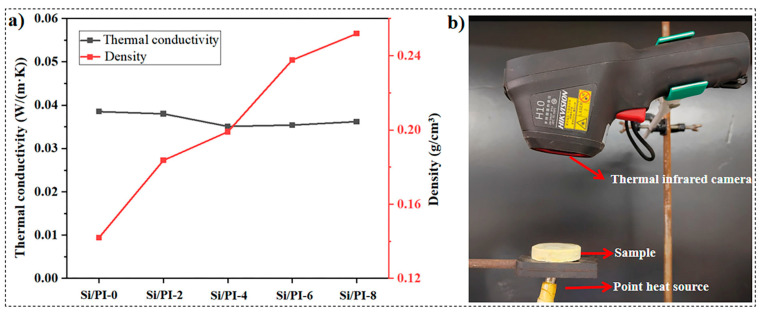
(**a**) Density and thermal conductivity of the samples. (**b**) Experimental setup for thermal infrared testing.

Thermal conductivity, a fundamental parameter in the theory of heat conduction, stands as one of the most crucial indicators for evaluating the thermal insulation performance of porous materials. The internal heat conduction processes in porous materials encompass three primary modes: thermal radiation, gaseous conduction, and solid conduction [37]. Notably, at room temperature, the influence of thermal radiation can be disregarded [38]. Solid conduction primarily hinges on the material’s intrinsic properties, whereas gaseous conduction is contingent upon the material’s microstructure. For materials of similar composition, as density diminishes, so does the solid thermal conductivity. When the pore size of high-porosity materials shrinks below the mean free path of air molecules (approximately 70 nanometers), gaseous conduction becomes constrained. The pure silica aerogel prepared here boasts a density of 142 kg/m^3^ and a thermal conductivity of 0.039 W/(m·K) (Figure 5a). Intriguingly, despite an increase in overall density with the incorporation of additional PI, the thermal conductivity coefficient of the material actually decreases rather than increases. This phenomenon arises because the polyimide gel infused into the silica skeleton displays a typical mesoporous structure (smaller than the mean free path of air, which is approximately 70 nm, as depicted in Figure S3). This structure effectively mitigates gaseous conduction [39,40]. As a result, as the concentration of PAA rises (within the range of 20–80 kg/m^3^), the thermal conductivity coefficient of the composite material decreases from 0.039 W/(m·K) to approximately 0.035 W/(m·K).

**Figure 6:**
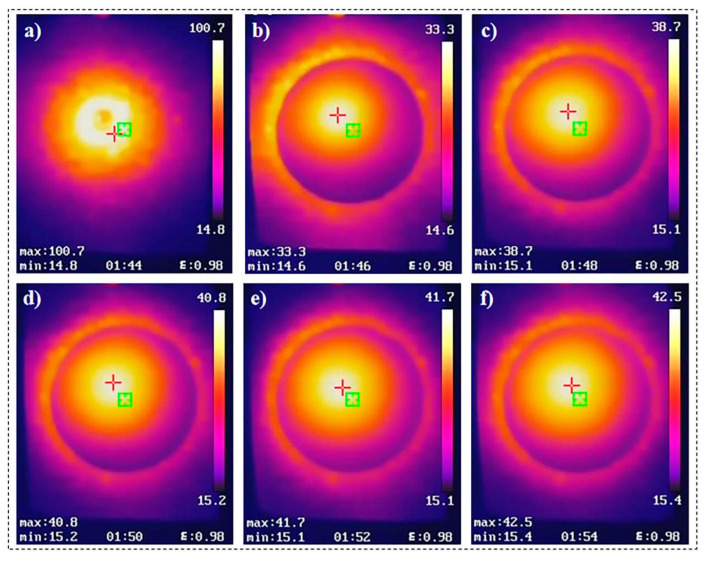
Infrared images of the Si/PI-6 aerogel captured at different time points during the thermal infrared imaging experiment. (**a**–**f**) Thermal infrared images captured at 0, 2, 4, 6, 8, and 10 min, respectively. The red “+” and green boxes in the figure represent the highest temperature point and the temperature collection area, respectively.

The thermal insulation performance of the composite material was investigated using an infrared thermography camera. The sample was positioned directly above and in contact with a point heat source maintained at a temperature of approximately 100 ± 1 °C. The infrared thermography camera was directed at the upper surface of the sample for observation, as illustrated in Figure 5b. The bottom of the sample was heated, and the temperature of the upper surface was recorded at two-minute intervals. After 10 min, the front surface temperature of Si/PI-6 had risen to 42 °C (Figure 6), which was lower than the surface temperature of pure silica aerogel, which reached 45.2 °C (Figure S8). When comparing the changes in the upper surface temperature of the two samples over the 10 min heating period (Figure S9), it was consistently observed that the upper surface temperature of the composite material remained lower than that of pure silica aerogel. This further confirms that the incorporation of PI enhances the material’s thermal insulation performance.

### 2.4. Flame Retardancy

**Figure 7:**
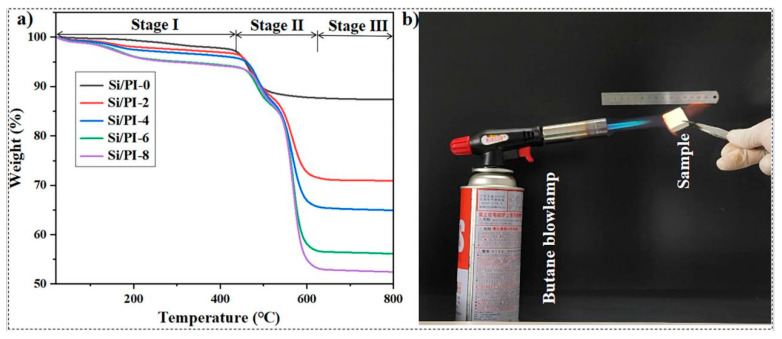
(**a**) TGA curves (measured in air) and (**b**) experimental setup for combustion testing of samples.

To investigate the high-temperature thermal stability of the composite material, thermogravimetric analysis (TGA) and ignition tests were conducted on the samples. The TGA curve of the sample, shown in Figure 7a, reveals the thermal decomposition process of the material. This process can be divided into three stages: 20–420 °C, 420–630 °C, and 630–800 °C. In the first stage (20–420 °C), the weight loss is primarily attributed to the volatilization of components: water vapor evaporating from the pores and weakly bonded volatile species from the material surface, such as residual solvent. The second stage (420–630 °C) exhibits the largest weight loss, primarily due to two factors. Firstly, the silica aerogel surface contains a large number of -Si-CH_3_ groups, which undergo thermal decomposition when the temperature exceeds 420 °C [41]. Additionally, in the composite aerogel, as the PI content increases, the initial temperature of thermal decomposition of the sample rises from 400 °C to 450 °C, and the final temperature of thermal decomposition increases from 530 °C to above 600 °C. This is mainly due to the excellent high-temperature stability of PI. In the third stage (630–800 °C), the weight loss is no longer significant, indicating that the methoxy groups and PI have been almost completely decomposed. Furthermore, in this stage, the PI molecular chains undergo complete thermal decomposition above 630 °C. Therefore, the higher the PI proportion in the material, the more pronounced the final weight loss of the composite material. The TGA data indicate that the introduction of PI can enhance the thermal stability of the silica aerogel. As the PI content increases, the thermal stability of the composite aerogel also gradually improves.

The experiment investigated the combustion behavior of the materials. The samples were exposed to the direct flame of an open butane torch (approximately 1300 °C), as illustrated in Figure 7. During the experiment, a cubic sample of Si/PI-6, with side lengths of 30 mm and a thickness of 10 mm, was placed 10 cm in front of the torch head, ensuring that the outer flame could directly aim at the center of the material for burning. When Si/PI-0 and Si/PI-6 were simultaneously removed from the flame, the former continued to burn until it was completely consumed. This was due to the large amount of Si-CH_3_ present in the material, which supported the combustion, allowing it to sustain burning even after being removed from the flame (see Supplementary Movie S1). In contrast, the Si/PI-6 composite material extinguished automatically (see Supplementary Movie S2). This is because PI molecules are characterized by a substantial content of heat-resistant benzene rings and aromatic imide groups. These functional groups exhibit remarkable char-forming capabilities during combustion, leading to superior flame retardancy. Structurally, in the pomegranate-like structured composite, the polyimide layers acted as barriers around the easily combustible silica skeleton, inhibiting the diffusion of thermal decomposition products and oxygen, thereby suppressing combustion. Additionally, a disk-shaped sample of Si/PI-6, with a diameter of 37 mm and a thickness of 10 mm, was placed 5 cm in front of the torch head, ensuring that the outer flame could directly aim at the center of the material for burning, as shown in Figure S10a. After burning for 20 min, the flame was extinguished and the sample was removed for observation. Figure S10b displays the morphology of the burned surface of the sample, which exhibited significant carbonization. A small number of cracks appeared on the burned surface, resulting from stress concentration in the silicon dioxide particles within the sample during the burning process. However, there was no detachment of the burned layer, and the sample maintained its structural integrity after burning. The back of the sample retained its original pale yellow color, as shown in Figure S10c, indicating that after 20 min of high-temperature burning, the back of the material did not undergo high-temperature oxidation discoloration. This demonstrates the material’s excellent high-temperature thermal insulation performance. High-temperature thermal stability enables the material to maintain stable performance under more extreme temperature conditions.

### 2.5. Hydrophobicity

**Figure 8:**
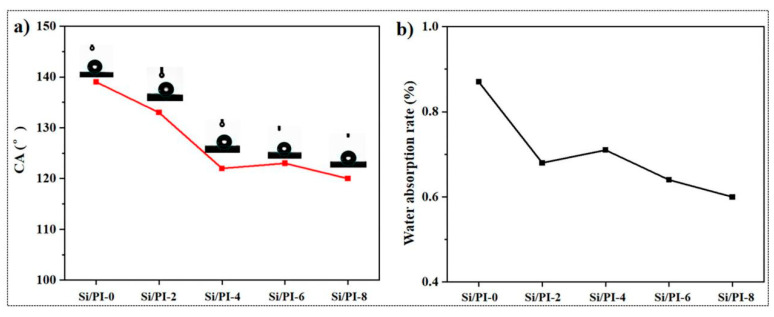
(**a**) WCAs of the samples. (**b**) Water absorption rate curve of the samples.

Hydrophobicity is a significant characteristic of thermal insulation materials [42]. The wettability of the composite aerogel has been comprehensively characterized. As shown in Figure 8a, all samples exhibited excellent hydrophobicity, with water contact angles (WCAs) exceeding 120°. According to the characterization results, pure silica aerogel exhibited the highest WCAs, reaching 138°. As the proportion of polyimide increased, the hydrophobicity of the material decreased but remained above 120°. To further verify the moisture resistance of the materials, all samples were exposed to saturated water vapor at 25 °C for 24 h to obtain water absorption curves (Figure 8b). The water absorption rates of all samples were below 1%, demonstrating excellent moisture resistance. Notably, the pure silica aerogel sample had the highest water absorption rate among the tested samples, at 0.87%. This can be attributed to the larger pore size of the silica aerogel, which favors capillary effects. The introduction of PI nanofibers filled the pores of the silica aerogel, reducing the water absorption capacity of the composites. As the amount of polyimide introduced increased, the water absorption rate of the composites decreased. Specifically, the water absorption rate of Si/PI-8 was only 0.53%. In summary, the composites with a “pomegranate-like” structure not only retained the excellent hydrophobicity of the silica aerogel material but also enhanced its moisture resistance. Therefore, the obtained composites are promising for applications in high-humidity environments.

## 3. Materials and Methods

### 3.1. Materials

The reagents, including methyltriethoxysilane (MTES, ≥98%), acetic acid (AR grade), sodium hydroxide (NaOH, AR grade), ethylene glycol (≥99%), N-methyl-2-pyrrolidone (NMP, 98%), pyridine (>99%), 1,2,4,5-benzenetetracarboxylic anhydride (PMDA, 99%), and 2,2′-bis(trifluoromethyl)benzidine (TFMB, 98%), were provided by Macklin. The dehydrating agent acetic anhydride (AR grade) was purchased from Sinopharm Chemical Reagent Co., Ltd., (Shanghai, China), The cross-linker 1,3,5-tris(4-aminophenoxy)benzene (TAB, ≥98%) was purchased from Aladdin. All these chemicals were used as received without further purification. Deionized water was used throughout the experiment to prepare all mixed solutions.

### 3.2. Methods

(1) Initially, 6 mL of methyltriethoxysilane monomer, 22.4 mL of water, and 0.2 mL of a 1 wt.% hydrochloric acid solution were mixed at room temperature and magnetically stirred for 30 min. Subsequently, a sodium hydroxide solution (1 mol/L) was added while stirring to adjust the alkalinity of the solution to a pH of 9, at which point the system formed a gel. The sample was then aged at 50 °C for 4 h to obtain a wet gel. The wet gel was submerged in a mixture of tert-butanol and water for solvent exchange three times, with each exchange lasting 24 h. Finally, the wet gel underwent freeze-drying for 24 h to obtain the silica aerogel.

(2) The polymerization of the polyimide was carried out through a two-step method. The reaction pathway for synthesizing polyimides is shown in Figure S1. Initially, 1,2,4,5-Benzenetetracarboxylic dianhydride (PMDA) and 2,2′-bis(trifluoromethyl) biphenylamine (TFMB) were added to an N-methyl-2-pyrrolidone (NMP) solvent at a molar ratio of 1.05:1, with the solid content in the solution controlled at 0.13 g/cm*^3^*. After vigorously stirring magnetically at room temperature for 2 h, a certain amount of the cross-linking agent 1,3,5-tris(4-aminophenoxy)benzene (TAB) was added to the solution, controlling the molar ratio between TAB and TFMB at 1:40. After polymerizing at room temperature for 30 min, a certain amount of acetic anhydride and pyridine (both in a molar ratio of 8:1 with respect to TFMB) were added, and the solution was diluted to the desired concentration. All the raw material ratios of polyimide corresponding to different samples are listed in Table 1. The previously prepared silica aerogel was immersed in this solution via isovolumetric impregnation and subsequently transferred to a sealed container, where it was allowed to stand for 24 h to form a composite gel. The composite gel was submerged in a mixture of tert-butanol and water (1:1 (*v*/*v*)) for solvent exchange three times, with each exchange lasting 24 h. Finally, the wet gel underwent freeze-drying for 24 h to obtain the composite samples. The silica/polyimide aerogel composites prepared through adjusting the concentration of the polyimide precursor are defined as Si/PI-x. Here, x represents 0, 2, 4, 6, and 8, corresponding to the concentrations of the polyimide precursor solution (x = 0, 0.02, 0.04, 0.06, and 0.08 g/cm^3^), respectively.

### 3.3. Characterization

The morphology and structure of the samples were characterized using Field Emission Scanning Electron Microscopy (SEM; model: Thermo Scientific Apreo 2C, produced by Thermo Fisher Scientific in Waltham, MA, USA). This SEM was equipped with an OXFORD ULTIM Max65 Energy Dispersive X-ray Spectrometer (produced by Oxford Instruments in the UK) for elemental mapping. Fourier Transform Infrared Spectroscopy (FTIR) was recorded using a Nicolet iS10 spectrometer, produced by Thermo Fisher Scientific in Waltham, MA, USA. All spectra were obtained by scanning each sample 16 times within the range of 1800 to 600 cm^−1^, at a resolution of 4 cm^−1^. The crystal structure was measured using an Ultima IV X-ray Diffractometer, produced by Rigaku Corporation in Tokyo, Japan, within the range of 5–30° (2θ). The mechanical compressive properties of the samples were measured using a WDW-D1000N Universal Testing Machine, produced by Jinan Xinguang Testing Machine Manufacturing Co., Ltd. in Jinan, China. The strain rate during testing was limited to 5 mm/min. The thermal conductivity of the samples was evaluated using a C-Therm TCi Thermal Conductivity Analyzer, produced in Fredericton, NB, Canada, through the transient plane source method. The thermal decomposition behavior of the samples in air was studied using a TGA5500 Thermogravimetric Analyzer, produced in the New Castle, DE, USA. The samples were placed in ceramic crucibles and heated from 20 °C to 800 °C at a heating rate of 10 °C/min. The static contact angle of the samples was measured at room temperature using an OCA 50 Contact Angle Measurement System, produced by Data Physics in Stuttgart, Germany. At least three specimens were tested for each sample, and the average value was reported. Infrared imaging was performed using an H10 Thermal Infrared Camera, produced by Hangzhou Hikmicro Sensing Technology Co., Ltd. in Hangzhou, China, which was aimed at the sample for capturing images.

## 4. Conclusions

To overcome the brittleness of silica aerogels, we successfully introduced polyimide into the silica system using the in situ gelation method, synthesizing a high-strength and flame-retardant silica/polyimide composite aerogel. The three-dimensional layers composed of polyimide nanofibers firmly encapsulate the silica microspheres, forming a “pomegranate-like” structure. The polyimide nanofiber layers serve as both an adhesive and a skeletal support, enabling the composite to maintain stability in both shape and structure. Furthermore, the numerous hydrogen bonds present at the interface between the two phases enhance the chemical compatibility of the interface and strengthen the interaction between them, imparting high mechanical strength to the composite and addressing the brittleness issue of silica aerogels. Additionally, the composite sample Si/PI-6 retains its structural integrity even after being subjected to high-temperature burning for 20 min. The pomegranate-like structured aerogel exhibits excellent overall performance and is capable of reducing energy consumption, making it a promising candidate for a wide range of applications in the field of thermal insulation.

## Figures and Tables

**Table 1:** Raw material ratios of polyimide corresponding to different samples.

Samples	PMDA (g)	TFMB (g)	TAB (g)	Acetic Anhydride (mL)	Pyridine (mL)	NMP (mL)
Si/PI-0	0	0	0	0	0	0
Si/PI-2	0.231	0.32	0.01	0.75	0.64	24.86
Si/PI-4	0.231	0.32	0.01	0.75	0.64	11.74
Si/PI-6	0.231	0.32	0.01	0.75	0.64	7.36
Si/PI-8	0.231	0.32	0.01	0.75	0.64	5.17

## Data Availability

The data presented in this study are available on request from the corresponding author.

## Supplementary Materials

The following supporting information can be downloaded at: https://www.mdpi.com/article/10.3390/molecules30081709/s1, Movie S1: Movie of the combustion process of Si/PI-0 aerogel.; Movie S2: Movie of the combustion process of Si/PI-6 aerogel; Figure S1. Reaction pathway for PI polymerization via two-step method; Figure S2. SEM image of Si/PI-6; Figure S3. SEM image of samples: (a) Si/PI-2, (b) Si/PI-4, (c) Si/PI-6, and (d) Si/PI-8; Figure S4. FTIR of samples: (a) Si/PI-0, (b) Si/PI-2, (c) Si/PI-4, (d) Si/PI-6, and (e) Si/PI-8; Figure S5. XRD of Si/PI-0 and Si/PI-6 samples; Figure S6. Stress–strain (σ–ε) curves and their corresponding linear regression curves for the samples: (a) Si/PI-0, (b) Si/PI-2, (c) Si/PI-4, (d) Si/PI-6, and (e) Si/PI-8; Figure S7. Yield properties (yield strength σ 0.2 and yield strain ɛ 0.2) of the samples: (a) Si/PI-0, (b) Si/PI-2, (c) Si/PI-4, (d) Si/PI-6, and (e) Si/PI-8; Figure S8. Infrared images of the Si/PI-0 at a point heat source of 100 °C; Figure S9. Comparison of heat transfer properties of Si/PI-0 and Si/PI-6 aerogels under a point heat source at 100 °C; Figure S10. Fire-retardant property of Si/PI-6 aerogel subjected to a butane lamp flame.

## References

[1] Kistler S.S. (1931). Coherent expanded aerogels and jellies. Nature.

[2] Guo C.N., Huang D.M., Lin P. (2017). Aggregate structural changes in silica aerogels with temperature. Emerg. Mater. Res..

[3] Meador M.A.B., Malow E.J., Silva R., Wright S., Quade D., Vivod S.L., Guo H.Q., Guo J., Cakmak M. (2012). Mechanically Strong, Flexible Polyimide Aerogels Cross-Linked with Aromatic Triamine. ACS Appl. Mater. Interfaces.

[4] Sun H.Y., Xu Z., Gao C. (2013). Multifunctional, ultra-flyweight, synergistically assembled carbon aerogels. Adv. Mater..

[5] Huang D.M., Guo C.N., Zhang M.Z., Shi L. (2017). Characteristics of nanoporous silica aerogel under high temperature from 950 °C to 1200 °C. Mater. Des..

[6] Pisal A.A., Rao A.V. (2016). Comparative studies on the physical properties of TEOS, TMOS and Na_2_SiO_3_ based silica aerogels by ambient pressure drying method. J. Porous Mater..

[7] He S., Li Z., Shi X.J., Yang H., Gong L.L., Cheng X.D. (2015). Rapid synthesis of sodium silicate based hydrophobic silica aerogel granules with large surface area. Adv. Powder Technol..

[8] Zhang R.B., An Z.M., Zhao Y., Zhang L., Zhou P. (2020). Nanofibers reinforced silica aerogel composites having flexibility and ultra-low thermal conductivity. Int. J. Appl. Ceram. Technol..

[9] Pierre A.C., Pajonk G.M. (2002). Chemistry of aerogels and their applications. Chem. Rev..

[10] Niu Z.W., He X.Y., Huang T., Tang B.C., Cheng X., Zhang Y., Shao Z.D. (2019). A facile preparation of transparent methyltriethoxysilane based silica xerogel monoliths at ambient pressure drying. Microporous Mesoporous Mater..

[11] Wang L.B., Guo R.L., Ren J.F., Song G.M., Chen G.X., Zhou Z., Li Q.F. (2020). Preparation of superhydrophobic and flexible polysiloxane aerogel. Ceram. Int..

[12] Rezaei S., Zolali A.M., Jalali A., Park C.B. (2020). Novel and simple design of nanostructured, super-insulative and flexible hybrid silica aerogel with a new macromolecular polyether-based precursor. J. Colloid Interface Sci..

[13] Cho J., Jang H.G., Kim S.Y., Yang B. (2019). Flexible and coatable insulating silica aerogel/polyurethane composites via soft segment control. Compos. Sci. Technol..

[14] Li H.M., Li J.H., Thomas A., Liao Y.Z. (2019). Ultra-High Surface Area Nitrogen-Doped Carbon Aerogels Derived from a Schiff-Base Porous Organic Polymer Aerogel for CO_2_ Storage and Supercapacitors. Adv. Funct. Mater..

[15] Yu Z.L., Yang N., Apostolopoulou-Kalkavoura V., Qin B., Ma Z.Y., Xing W.Y., Qiao C., Bergström L., Antonietti M., Yu S.H. (2018). Fire-Retardant and Thermally Insulating Phenolic-Silica Aerogels. Angew. Chem. Int. Ed..

[16] Li Z., Gong L.L., Cheng X.D., He S., Li C.C., Zhang H.P. (2016). Flexible silica aerogel composites strengthened with aramid fibers and their thermal behavior. Mater. Des..

[17] Song Q.Q., Miao C.Q., Sai H.Z., Gu J., Wang M.J., Jiang P.J., Wang Y.T., Fu R., Wang Y.X. (2022). Silica-bacterial cellulose composite aerogel fibers with excellent mechanical properties from sodium silicate precursor. Gels.

[18] Li C.D., Liu Q.S., Zhang G.H., Lin L.L., Ostrikov K. (2023). Rapid synthesis of MTES-derived silica aerogel monoliths in Cetyltrimethylammonium bromide/water solvent system by ambient pressure drying. Powder Technol..

[19] Li K.W., He S., Du C.H., Guo S.P., Huang Y.J. (2024). Ultra flexible silica aerogel with excellent mechanical properties for durable oil-water separation. J. Environ. Chem. Eng..

[20] Li Z., Cheng X.D., Shi L., He S., Gong L.L., Li C.C., Zhang H.P. (2016). Flammability and oxidation kinetics of hydrophobic silica aerogels. J. Hazard. Mater..

[21] Zheng Z., Zhao Y.L., Hu J.H., Wang H.T. (2020). Flexible, Strong, Multifunctional Graphene Oxide/Silica-Based Composite Aerogels via a Double-Cross-Linked Network Approach. ACS Appl. Mater. Interfaces.

[22] Patil S.P. (2021). Enhanced mechanical properties of double-walled carbon nanotubes reinforced silica aerogels: An all-atom simulation study. Scr. Mater..

[23] Slosarczyk A. (2017). Recent Advances in Research on the Synthetic Fiber Based Silica Aerogel Nanocomposites. Nanomaterials.

[24] Zhang T., Yu D.P., Xu F.H., Kong Y., Shen X.D. (2024). Flexible Silica aerogel composites for Thermal Insulation under High-Temperature and Thermal-Force Coupling Conditions. ACS Appl. Nano Mater..

[25] Zhu C.Y., Xue T.T., Ma Z.C., Fan W., Liu T.X. (2023). Mechanically Strong and Thermally Insulating Polyimide Aerogel Fibers Reinforced by Prefabricated Long Polyimide Fibers. ACS Appl. Mater. Interfaces.

[26] Zhang X.D., Yang J., Cheng Y.L., Zhao S.P., Fan J.P. (2024). Elastic, strong polyimide/boron oxide composite aerogel with high thermal stability properties. Mater. Lett..

[27] Liu T., Liang F.W., Chen S., Zhang P., Qian K., Xu Y., Guo W.W. (2023). Aramid reinforced polyimide aerogel composites with high-mechanical strength for thermal insulation material. Polym. Adv. Technol..

[28] Zhang S.Z., Liu C., Wang Z., Wang J., Xu G.Y., Shi H.W. (2024). Ultralow shrinkage polyimide hybrid composite aerogel enhanced with organic fibers for thermal protection. J. Appl. Polym. Sci..

[29] Xue T.T., Yu Y., Fu Z.P., Wang Q.Y., Hu Z.Y., Fan W., Liu T.X. (2023). Double-network polyimide/silica aerogel fiber for thermal insulation under extremely hot and humid environment. Compos. Sci. Technol..

[30] Zhang X.H., Ni X.X., Li C.X., You B., Sun G. (2020). Co-gel strategy for preparing hierarchically porous silica/polyimide nanocomposite aerogel with thermal insulation and flame retardancy. J. Mater. Chem. A.

[31] Ren J., Zhang T., Kong Y., Zhao Z.Y., Zhu K.M., Zhang X.Q., Shen X.D. (2023). Facile synthesis of phenolic-reinforced silica aerogel composites for thermal insulation under thermal-force coupling conditions. Ceram. Int..

[32] Xi S., Wang Y.J., Zhang X.X., Cao K.L., Su J., Shen J., Wang X.D. (2023). Fire-resistant polyimide-silica aerogel composite aerogels with low shrinkage, low density and high hydrophobicity for aerospace applications. Polym. Test..

[33] Wu Y.W., Ye M.F., Zhang W.C., Yang R.J. (2017). Polyimide Aerogels Crosslinked through Cyclic Ladder-like and Cage Polyamine Functionalized Polysilsesquioxanes. J. Appl. Polym. Sci..

[34] Gao R., Zhou Z.J., Zhang H.B., Zhang X.G., Wu Y.M. (2023). The Evolution of Insulation Performance of Fiber-Reinforced Silica Aerogel after High-Temperature Treatment. Materials.

[35] Salihi E.C., Zarrabi A., Zarepour A., Gürboğa M., Niar S.H.N., Özakpınar O.B., Wang J., Datan H., Khosravi A., Šiller L. (2025). Ambient pressure dried graphene oxide-silica composite aerogels as pharmaceutical nanocarriers. J. Sol-Gel Sci. Technol..

[36] Zhao L., Chen J.Y., Pan D.F., Hou Y. (2023). Robust, Fire-Retardant, and Water-Resistant Wood/Polyimide composite aerogels with a Hierarchical Pore Structure for Thermal Insulation. Gels.

[37] LU X., Arduinischuster M.C., Kuhn J., Nilsson O., Fricke J., Pekala R.W. (1992). Thermal conductivity of monolithic organic aerogels. Science.

[38] Hou Y., Chen J.Y., Pan D.F., Zhao L. (2023). Directional-Freezing-Assisted In Situ Sol-Gel Strategy to Synthesize High-Strength, Fire-Resistant, and Hydrophobic Wood-Based composite aerogels for Thermal Insulation. Gels.

[39] Li T., Song J.W., Zhao X.P., Yang Z., Pastel G., Xu S.M., Jia C., Dai J.Q., Chen C.J., Gong A. (2018). Anisotropic, lightweight, strong, and super thermally insulating nanowood with naturally aligned nanocellulose. Sci. Adv..

[40] Jelle B.P. (2011). state-of-the-art and future thermal building insulation materials and solutions-Properties, requirements and possibilities. Energ. Build..

[41] Gu J., Fu R., Kang S.C., Yang X., Song Q.Q., Miao C.Q., Ma M.H., Wang Y.X., Sai H. (2022). Robust composite aerogel beads with pomegranate-like structure for water-based thermal insulation coating. Constr. Build. Mater..

[42] Zhang J.Y., Cheng Y.H., Xu C.J., Gao M.Y., Zhu M.F., Jiang L. (2021). Hierarchical Interface Engineering for Advanced Nanocellulosic Hybrid Aerogels with High Compressibility and Multifunctionality. Adv. Funct. Mater..

