# Supplementary material for: Robust and Hydrophobic Silica/Polyimide Aerogel with Pomegranate-like Structure for Thermal Insulation and Flame Retardancy up to 1300 °C
Source: Molecules. 2025 Apr 11;30(8):1709. doi: 10.3390/molecules30081709 (PMC12029470; doi:10.3390/molecules30081709)
Supplement: Supplementary file 1 [file molecules-30-01709-s001.zip › Supplementary Materials.pdf]

Robust and Hydrophobic Silica/polyimide Aerogel with Pomegranate-like Structure for Thermal Insulation and Flame Retardancy up to 1300℃

Junyong Chen*，Defang Pan

Corresponding author: chenjunyong17@mails.ucas.ac.cn

This supplementary information contains:

1. Supplementary movies
2. Reaction pathway for PI polymerization reaction
3. SEM images of Si/PI-6 aerogel
4. SEM images of Si/PI aerogels
5. FT-IR of Si/PI aerogels
6. XRD of Si/PI-0 and Si/PI-6 aerogels
7. Stress-strain curves and corresponding linear regression curves of Si/PI aerogels
8. Yield properties of Si/PI aerogels
9. Infrared images of Si/PI-6 aerogel
10. Comparison of heat transfer properties of Si/PI-0 and Si/PI-6 aerogels
11. Fire-retardant property of Si/PI-6 aerogel

1. Supplementary Movies

Movie S1

Movie of the combustion process of Si/PI-0 aerogel.

Movie S2

Movie of the combustion process of Si/PI-6 aerogel.

2.Reaction Pathway for Polyimide Synthesis

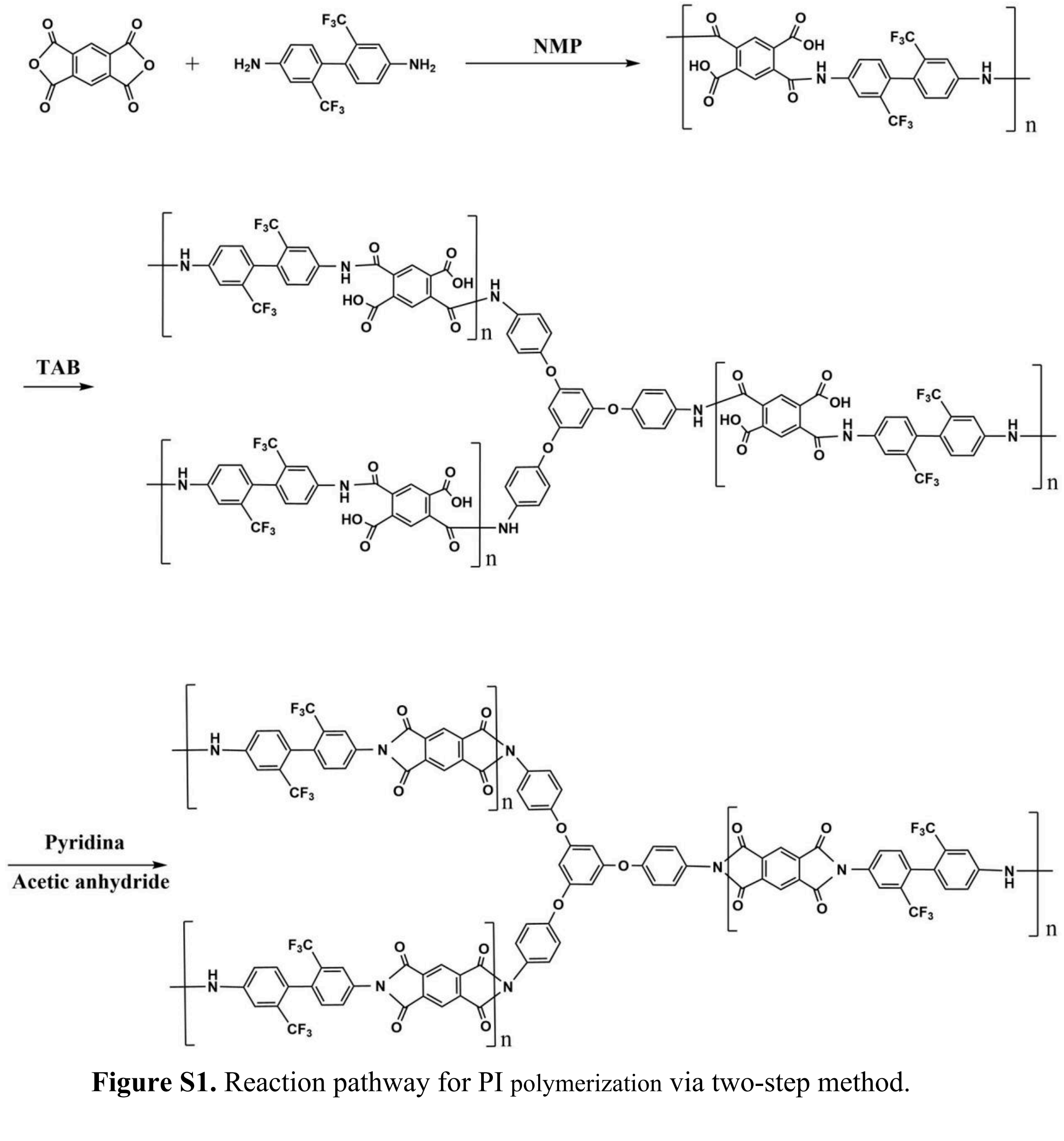

Figure S1. Reaction pathway for PI polymerization via two-step method.

3. SEM images of Si/PI-6 aerogel

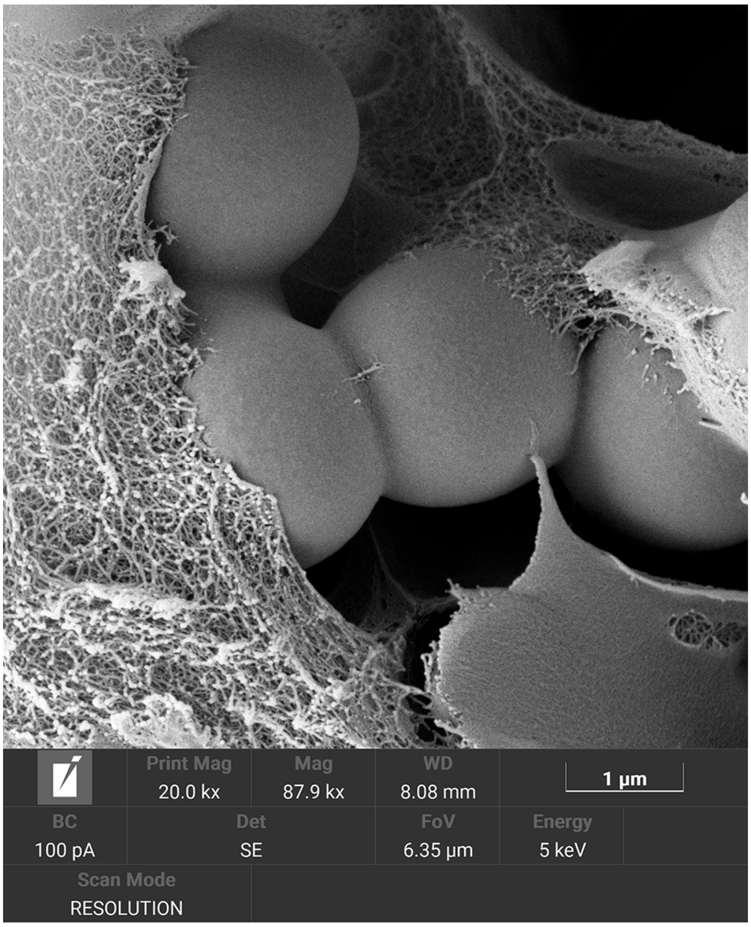

Figure S2. SEM image of Si/PI-6.

4. SEM images of Si/PI aerogels

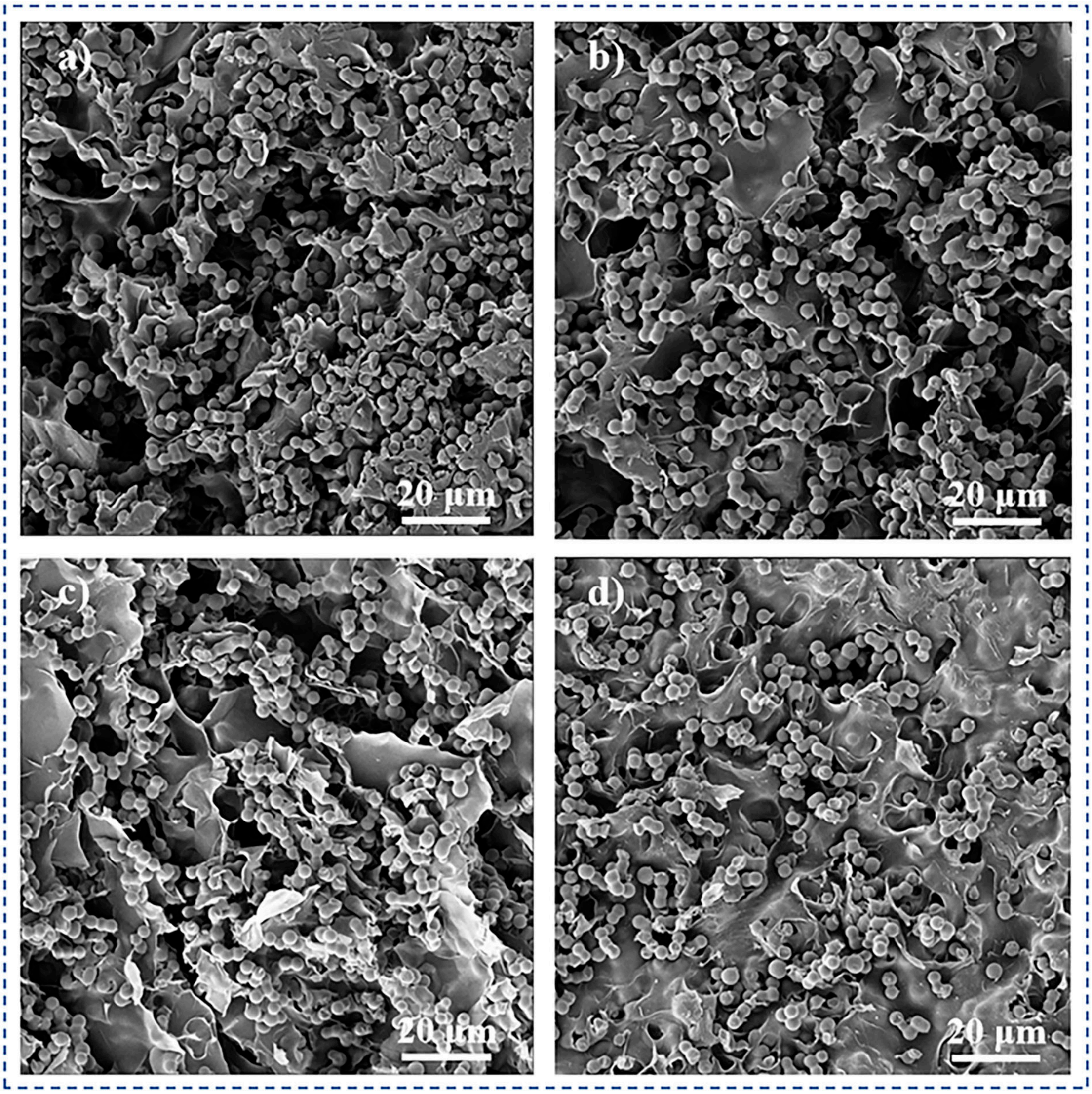

Figure S3. SEM image of samples. (a) Si/PI-2, (b) Si/PI-4, (c) Si/PI-6, (d) Si/PI-8.

5. FT-IR of Si/PI aerogels

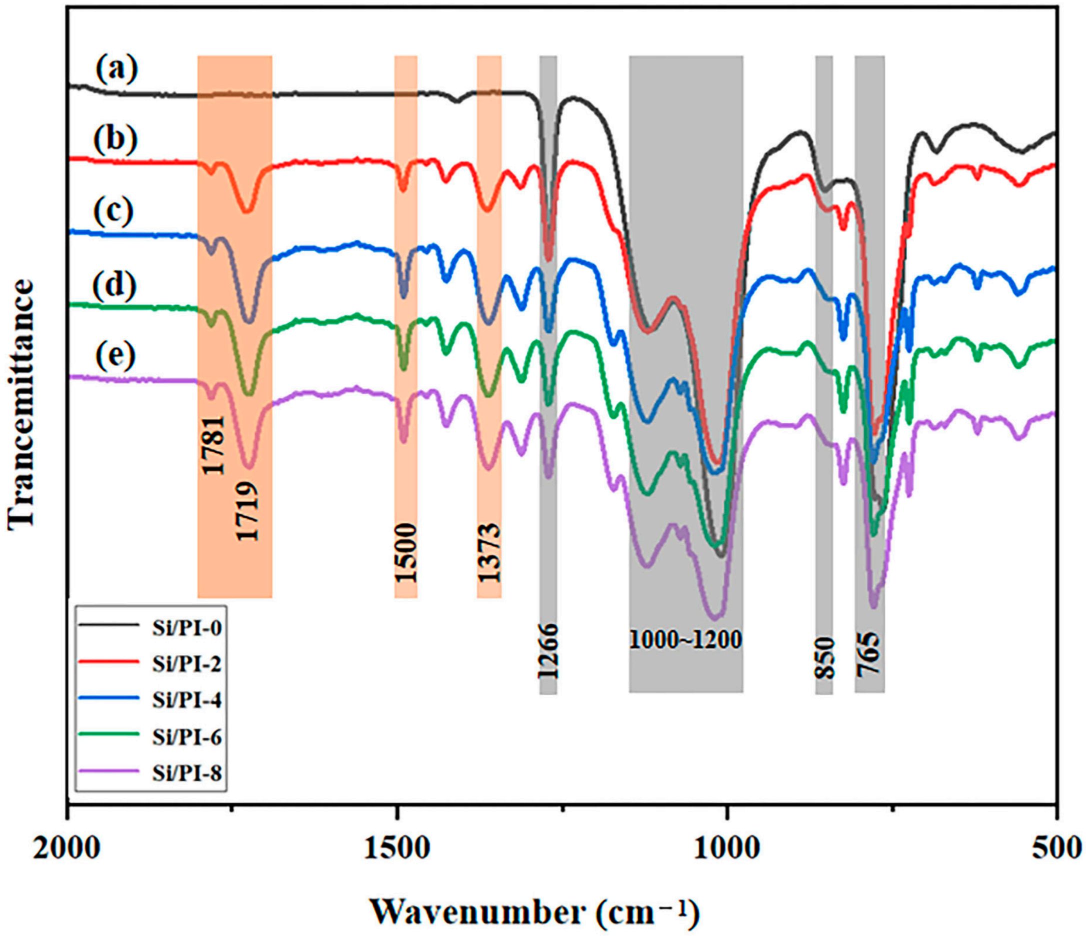

Figure S4. FT-IR of samples. (a) Si/PI-0, (b) Si/PI-2, (c) Si/PI-4, (d) Si/PI-6, (e) Si/PI-8.

6. XRD of Si/PI-0 and Si/PI-6 aerogels

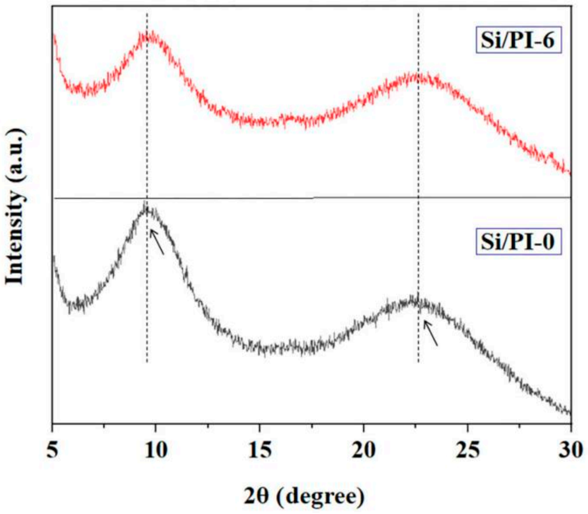

Figure S5. XRD of Si/PI-0 and Si/PI-6 samples.

7. Stress–strain (σ-ε) curves and corresponding linear regression curves of Si/PI aerogels

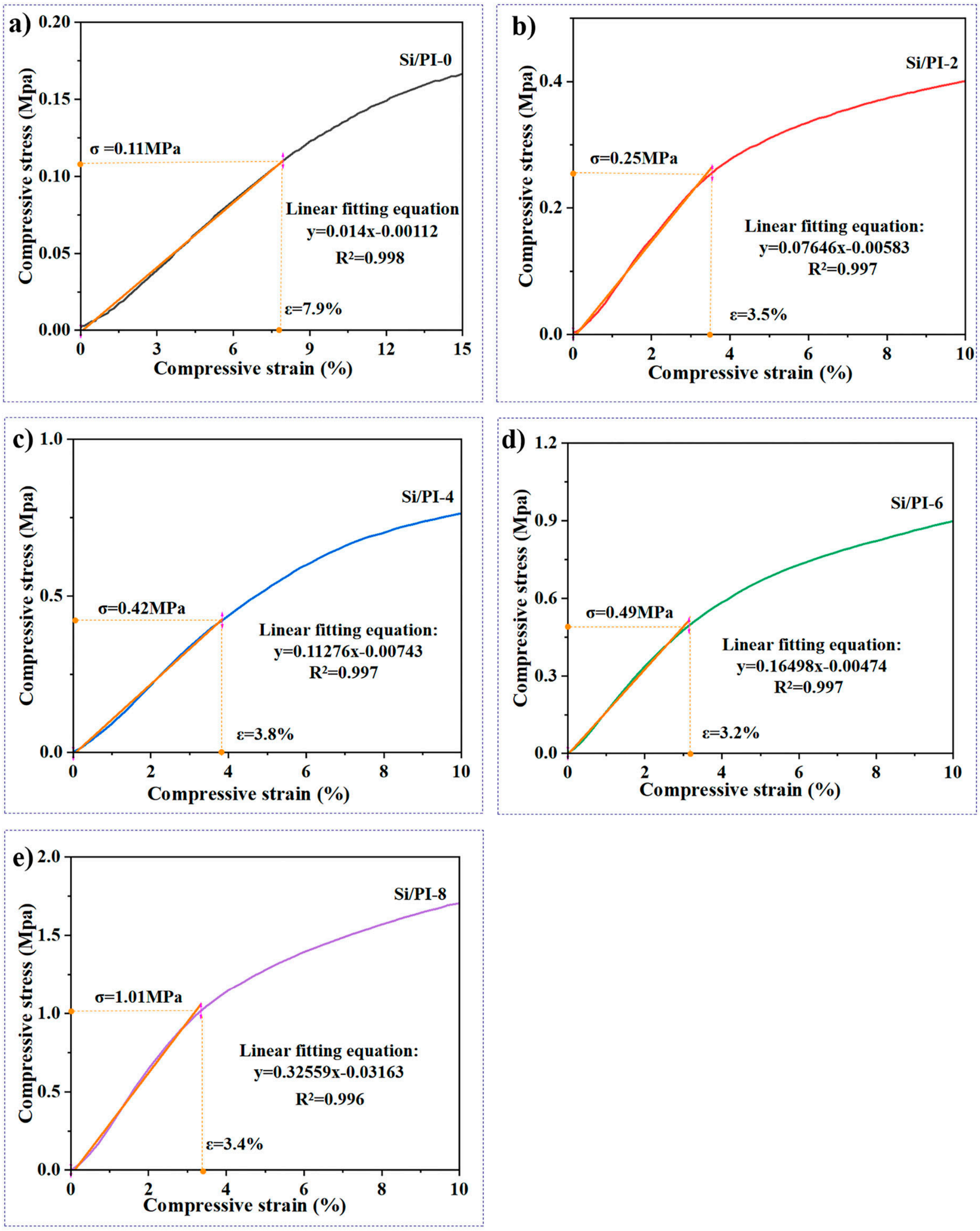

Figure S6. Stress–strain (σ-ε) curves and their corresponding linear regression curves for the samples: (a) Si/PI-0, (b) Si/PI-2, (c) Si/PI-4, (d) Si/PI-6, and (e) Si/PI-8.

8. Yield properties of Si/PI aerogels

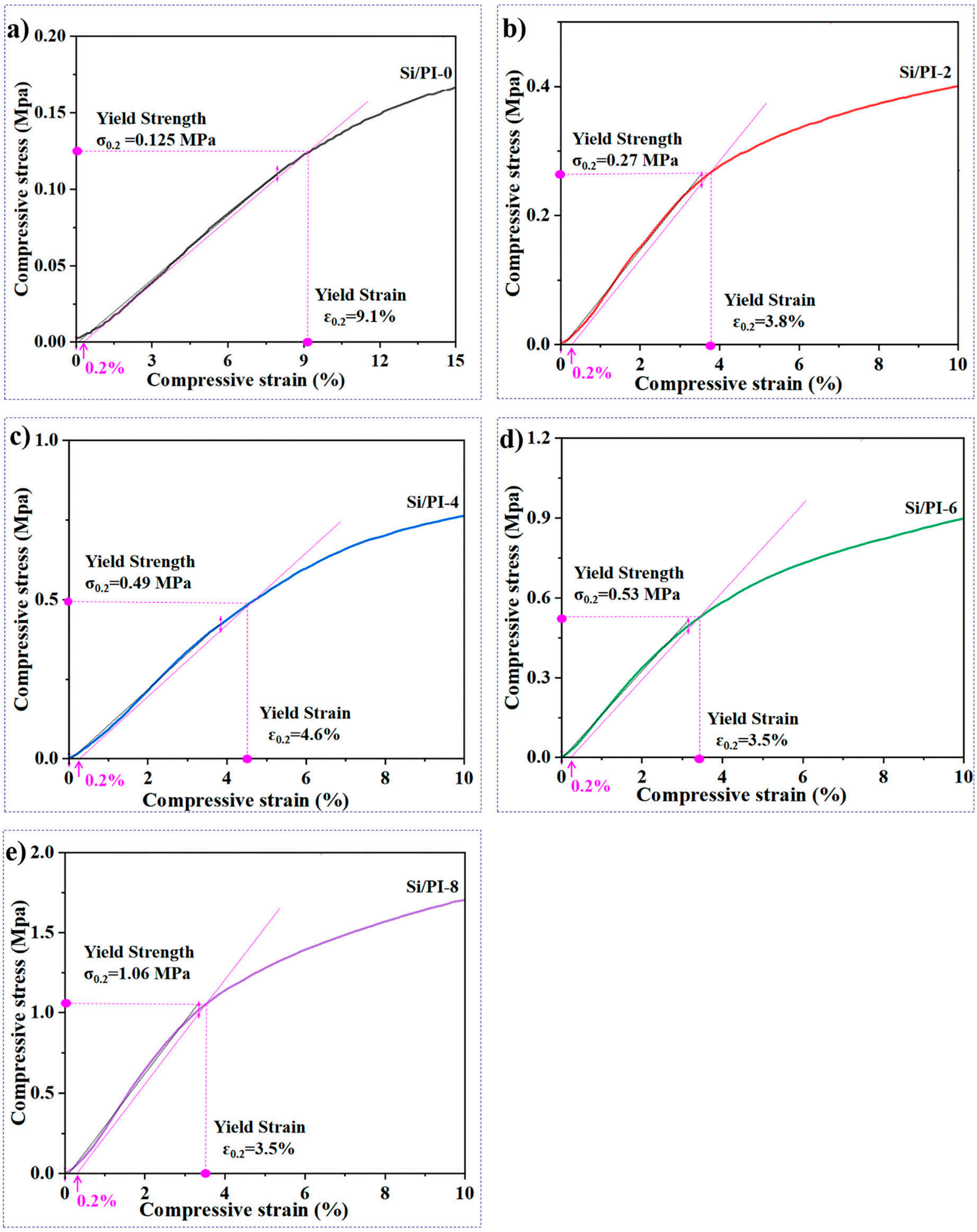

Figure S7. Yield properties (Yield Strength $\sigma_{0.2}$ and Yield Strain $\varepsilon_{0.2}$) of the samples: (a) Si/PI-0, (b) Si/PI-2, (c) Si/PI-4, (d) Si/PI-6, and (e) Si/PI-8.

9. Infrared images of Si/PI-6 aerogel

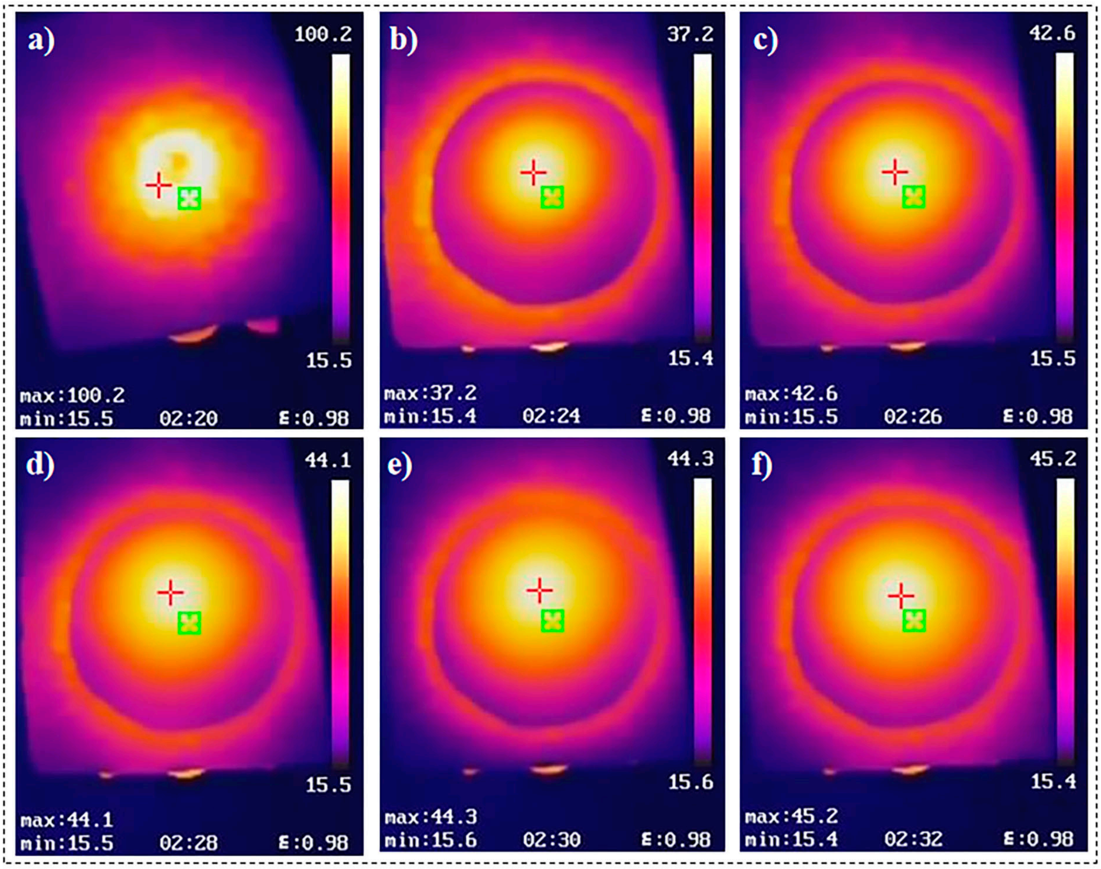

Figure S8. Infrared images of the Si/PI-0 at a point heat source of 100°C. (a–f) represent thermal infrared images captured at 0, 2, 4, 6, 8, and 10 min, respectively. The red "+" and green boxes in the figure represent the highest temperature point and the temperature collection area, respectively.

10. Comparison of heat transfer properties of Si/PI-0 and Si/PI-6 aerogels

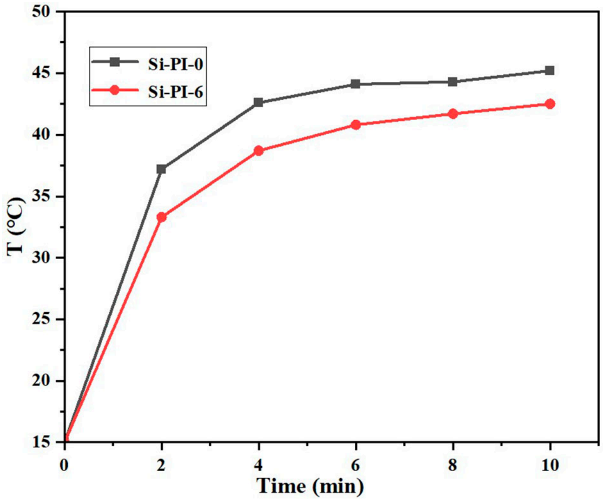

Figure S9. Comparison of heat transfer properties of Si/PI-0 and Si/PI-6 aerogels under a point heat source at 100°C.

11. Fire-retardant property of Si/PI-6 aerogel

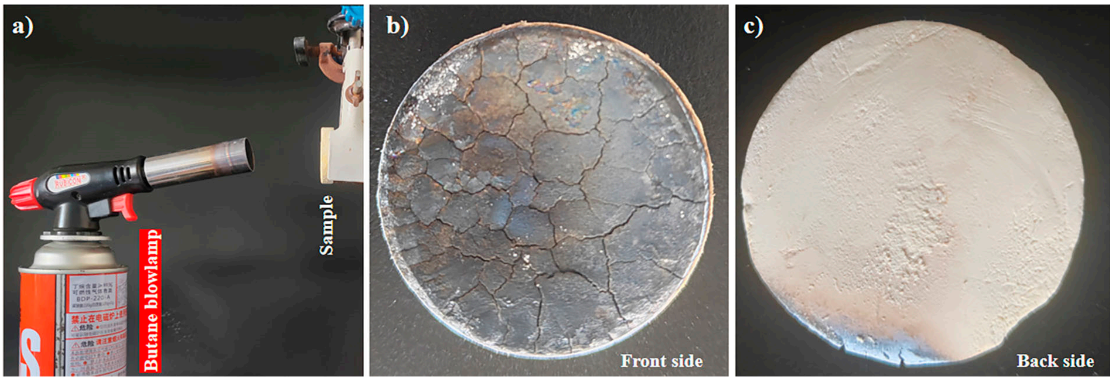

Figure S10. Fire-retardant property of Si/PI-6 aerogel subjected to an butane lamp flame. (a) Experimental setup for thermal infrared testing, (b) The post-burning morphology of the sample's combustion surface, (c) The post-burning morphology of the sample's back surface.
